# Supplementary material for: Combinative protein expression of immediate early genes c-Fos, Arc, and Npas4 along aversive- and reward-related neural networks
Source: bioRxiv. 2025 May 22:2025.04.21.649441. Preprint. [Version 3] doi: 10.1101/2025.04.21.649441 (PMC12139746; doi:10.1101/2025.04.21.649441)
Supplement: Supplement 1 [file NIHPP2025.04.21.649441v3-supplement-1.pdf]

bioRxiv preprint doi: https://doi.org/10.1101/2025.04.21.649441; this version posted May 22, 2025. The copyright holder for this preprint (which was not certified by peer review) is the author/funder, who has granted bioRxiv a license to display the preprint in perpetuity. It is made available under aCC-BY 4.0 International license.

Supplementary Figure Legends

Supp. Figure S1: IEG expression in PFC

(A, B) Larger field-of-view images of the PL (A) and IL (B). White dashed line indicates the region-of-interest (ROI) of each subregion used for automated cell detection analysis. Scale bars, 400 µm.

Supp. Figure S2: IEG expression in BLA

(A, B) Larger field-of-view images of the aBLA (A) and pBLA (B). White dashed line indicates the ROI of each subregion. Scale bars, 200 µm.

Supp. Figure S3: IEG expression in dDG

Larger field-of-view images of the dDG. White dashed line indicates the ROI. Scale bars, 200 µm. Uneven background was observed as the darker background level around subgranular zone of the granule cell layer in the Npas4 and Arc images.

Supp. Figure S4: IEG expression in vDG

Larger field-of-view images of the vDG. White dashed line indicates the ROI. Scale bars, 400 µm. Uneven background was observed in the c-Fos images of HC and CFC, and the Arc image of RC, as the increased autofluorescence along the granule cell layer.

Supp. Figure S5: IEG expression in aRSC

(A) Positions of the dorsal and ventral aRSC in the brain atlas (Allen Institute for Brain Science 2004). (B, C) Larger field-of-view images of the dorsal aRSC (B) and ventral aRSC (C). White dashed line indicates the ROI of each subregion. Scale bars, 400 µm.

Supp. Figure S6: IEG expression in pRSC

(A) Positions of the dorsal and ventral pRSC in the brain atlas (Allen Institute for Brain Science 2004). (B, C) Larger field-of-view images of the dorsal pRSC (B) and ventral pRSC (C). White dashed line indicates the ROI of each subregion. Scale bars, 400 µm.

Supp. Figure S7: Cell density and expression level of IEG-positive cells in PFC and BLA

(A–C), Analysis in the PL. (A) Cell density of c-Fos, Npas4, and Arc positive cells in HC, CFC, and RC in the PL. (B) Expression level of c-Fos, Npas4, and Arc positive cells in HC, CFC, and RC. n = 962, 4462, and 3897 cells for HC, CFC, and RC. (C) Percentage of c-Fos, Npas4, and Arc positive neurons per all $NeuN^+$ cells. (D–F), Analysis in the IL. (D) Cell density of c-Fos, Npas4, and Arc positive cells in HC, CFC, and RC in IL. (E) Expression level of c-Fos, Npas4, and Arc positive cells in HC, CFC, and RC. n = 1228, 5058, and 4935 cells for HC, CFC, and RC. (F) Percentage of c-Fos, Npas4, and Arc positive neurons per all $NeuN^+$ cells. (G–I), Analysis in the aBLA. (G) Cell density of c-Fos, Npas4, and Arc positive cells in HC, CFC, and RC in the aBLA. (H) Expression level of c-Fos, Npas4, and Arc positive cells in HC, CFC, and RC. n = 585, 987, and 1031 cells for HC, CFC, and RC. (I) Percentage of c-Fos, Npas4, and Arc positive neurons per all $NeuN^+$ cells. (J–L), Analysis in the pBLA. (J) Cell density of c-Fos, Npas4, and Arc positive cells in HC, CFC, and RC in pBLA. (K) Expression level of c-Fos, Npas4, and Arc positive cells in HC, CFC, and RC. n = 437, 1110, and 1133 cells for HC, CFC, and RC. (L) Percentage of c-Fos, Npas4, and Arc positive neurons per all $NeuN^+$ cells.

Supp. Figure S8: Cell density and expression of IEG-positive cells in DG and RSC

(A, B), Analysis in the dDG. (A) Cell density of c-Fos, Npas4, and Arc positive cells in HC, CFC, and RC in the dDG. (B) Expression level of c-Fos, Npas4, and Arc positive cells in HC, CFC, and RC. n = 1413, 1542, and 1628 cells for HC, CFC, and RC. (C, D), Analysis in the vDG. (C) Cell density of c-Fos, Npas4, and Arc positive cells in HC, CFC, and RC in vDG. (D) Expression level of c-Fos, Npas4, and Arc positive cells in HC, CFC, and RC. n = 749, 1406,

bioRxiv preprint doi: https://doi.org/10.1101/2025.04.21.649441; this version posted May 22, 2025. The copyright holder for this preprint (which was not certified by peer review) is the author/funder, who has granted bioRxiv a license to display the preprint in perpetuity. It is made available under aCC-BY 4.0 International license.

and 986 cells for HC, CFC, and RC. (E, F), Analysis in the dorsal aRSC. (E) Cell density of c-Fos, Npas4, and Arc positive cells in HC, CFC, and RC in the dorsal aRSC. (F) Expression level of c-Fos, Npas4, and Arc positive cells in HC, CFC, and RC. n = 783, 2103, and 2552 cells for HC, CFC, and RC. (G, H), Analysis in the ventral aRSC. (G) Cell density of c-Fos, Npas4, and Arc positive cells in HC, CFC, and RC in the ventral aRSC. (H) Expression level of c-Fos, Npas4, and Arc positive cells in HC, CFC, and RC. n = 1276, 2888, and 3068 cells for HC, CFC, and RC. (I, J), Analysis in dorsal pRSC. (I) Cell density of c-Fos, Npas4, and Arc positive cells in HC, CFC, and RC in the dorsal pRSC. (J) Expression level of c-Fos, Npas4, and Arc positive cells in HC, CFC, and RC. n = 1458, 2724, and 2102 cells for HC, CFC, and RC. (K, L), Analysis in the ventral pRSC. (K) Cell density of c-Fos, Npas4, and Arc positive cells in HC, CFC, and RC in the ventral pRSC. (L) Expression level of c-Fos, Npas4, and Arc positive cells in HC, CFC, and RC. n = 1955, 3931, and 4149 cells for HC, CFC, and RC.

Supp. Figure S9: Effect size of cell density and intensity
(A) Bar plots of Cohen's d of cell densities across brain regions, calculated from the data shown in Supp. Figure S7A, D, G, J and S8A, C, E, G, I, K. Gray dashed lines indicate the effects are small (d = ±0.2), medium (d = ±0.5), and large (d = ±0.8). (B) Bar plots of Cliff's delta of IEG intensities across brain regions, calculated from the data shown in Supp. Figure S7B, E, H, K and S8B, D, F, H, J, L. Gray dashed lines indicate the effects are small (δ = ±0.147), medium (δ = ±0.33), and large (δ = ±0.474).

Supp. Figure S10: Cell density changes in each IEG in different brain regions
(A) Scatter plots of fold-changes of c-Fos, Npas4, and Arc positive cell densities by CFC across ten brain regions, obtained from Figure 3A. (B) Similarly, scatter plots of fold-changes of c-Fos, Npas4, and Arc positive cell densities by RC, obtained from Figure 3A. Gray dashed lines indicate linear regression line. R and p indicate values of Pearson correlation.

Supp. Figure S11: Cell density ratio per all IEG positive cells in each cell group
Ratio of cell densities per all IEG positive cells. For each group of bars, the left bar indicates HC, the middle bar indicates CFC, and the right bar indicates RC groups. (A) PL, (B) IL, (C) aBLA, (D) pBLA, (E) dDG, (F) vDG, (G) dorsal aRSC, (H) ventral aRSC, (I) dorsal pRSC, and (J) ventral pRSC.

Supp. Figure S12: Intensities of IEGs in individual cells in PFC and BLA
Scatter plots showing the intensities of c-Fos, Npas4, and Arc in single cells. (A) PL, (B) IL, (C) aBLA, and (D) pBLA. Colors represent cells with selective or concurrent expression of c-Fos, Npas4, and Arc. Gray dashed lines indicate correlations of cells across all groups. Intensities within each cell group are shown in Supp. Fig. S15. Correlation coefficients in each cell group are shown in Supp. Fig. S18.

Supp. Figure S13: Intensities of IEGs in individual cells in DG
Scatter plots showing the intensities of c-Fos, Npas4, and Arc in single cells. (A) dDG and (B) vDG. Colors represent cells with selective or concurrent expression of c-Fos, Npas4, and Arc. Gray dashed lines indicate correlations of cells across all groups. Intensities within each cell group are shown in Supp. Fig. S16. Correlation coefficients in each cell group is shown in Supp. Fig. S19.

Supp. Figure S14: Intensities of IEGs in individual cells in RSC
Scatter plots showing the intensities of c-Fos, Npas4, and Arc in single cells. (A) dorsal aRSC, (B) ventral aRSC, (C) dorsal pRSC, and (D) ventral pRSC. Colors represent cells with selective or concurrent expression of c-Fos, Npas4, and Arc. Gray dashed lines indicate correlations of cells across all groups. Intensities within each cell group are shown in Supp. Fig. S17. Correlation coefficients in each cell group are shown in Supp. Fig. S20.

Supp. Figure. S15: Intensities of IEGs in each cell group in PFC and BLA

bioRxiv preprint doi: https://doi.org/10.1101/2025.04.21.649441; this version posted May 22, 2025. The copyright holder for this preprint (which was not certified by peer review) is the author/funder, who has granted bioRxiv a license to display the preprint in perpetuity. It is made available under aCC-BY 4.0 International license.

Intensities of c-Fos, Npas4, and Arc in individual cells in HC, CFC, and RC, in the PL (A), IL (B), aBLA (C), and pBLA (D).

Supp. Figure S16: Intensities of IEGs in each cell group in DG
Intensities of c-Fos, Npas4, and Arc in individual cells in HC, CFC, and RC, in the dDG (A) and vDG (B).

Supp. Figure S17: Intensities of IEGs in each cell group in RSC
Intensities of c-Fos, Npas4, and Arc in individual cells in HC, CFC, and RC, in the dorsal aRSC (A), ventral aRSC (B), dorsal pRSC (C), and ventral pRSC (D).

Supp. Figure S18: Correlation of IEG Intensities in each cell group in PFC and BLA
Average correlation of IEG intensities of c-Fos, Npas4, and Arc in individual cells in HC, CFC, and RC, in the PL (A), IL (B), aBLA (C), and pBLA (D).

Supp. Figure S19: Correlation of IEG Intensities in each cell group in DG
Average correlation of IEG intensities of c-Fos, Npas4, and Arc in individual cells in HC, CFC, and RC, in the dDG (A) and vDG (B).

Supp. Figure S20: Correlation of IEG Intensities in each cell group in RSC
Average correlation of IEG intensities of c-Fos, Npas4, and Arc in individual cells in HC, CFC, and RC, in the dorsal aRSC (A), ventral aRSC (B), dorsal pRSC (C), and ventral pRSC (D).

Supp. Figure Fig. S21: Functional connectivity network of IEG overlapping cells
(A–D) Similarly to Figure 8, Inter-regional correlation matrices and connectivity networks based on c-Fos$^+$/Npas4$^+$ (A), c-Fos$^+$/Arc$^+$ (B), Npas4$^+$/Arc$^+$ (C), and c-Fos$^+$/Npas4$^+$/Arc$^+$ cells (D). (E, F) Quantification of network complexity: Average number of edges per node (E) and Average number of edges per effective node (F). (G) Average of absolute correlation values in the correlation matrices for each cell group which are shown in Figure 8A–C and S21A–D.

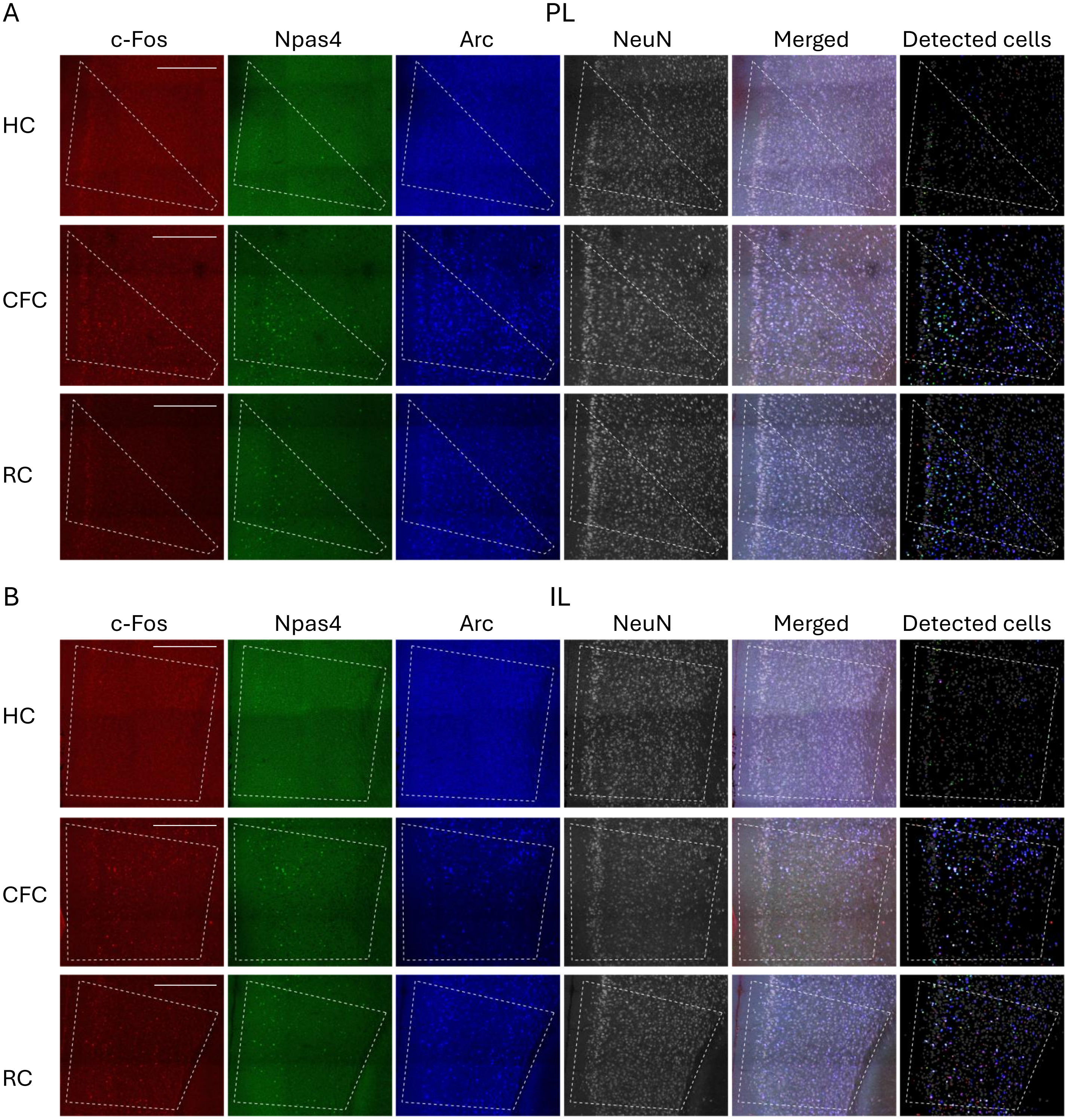

Supp. Figure S1: IEG expression in PFC

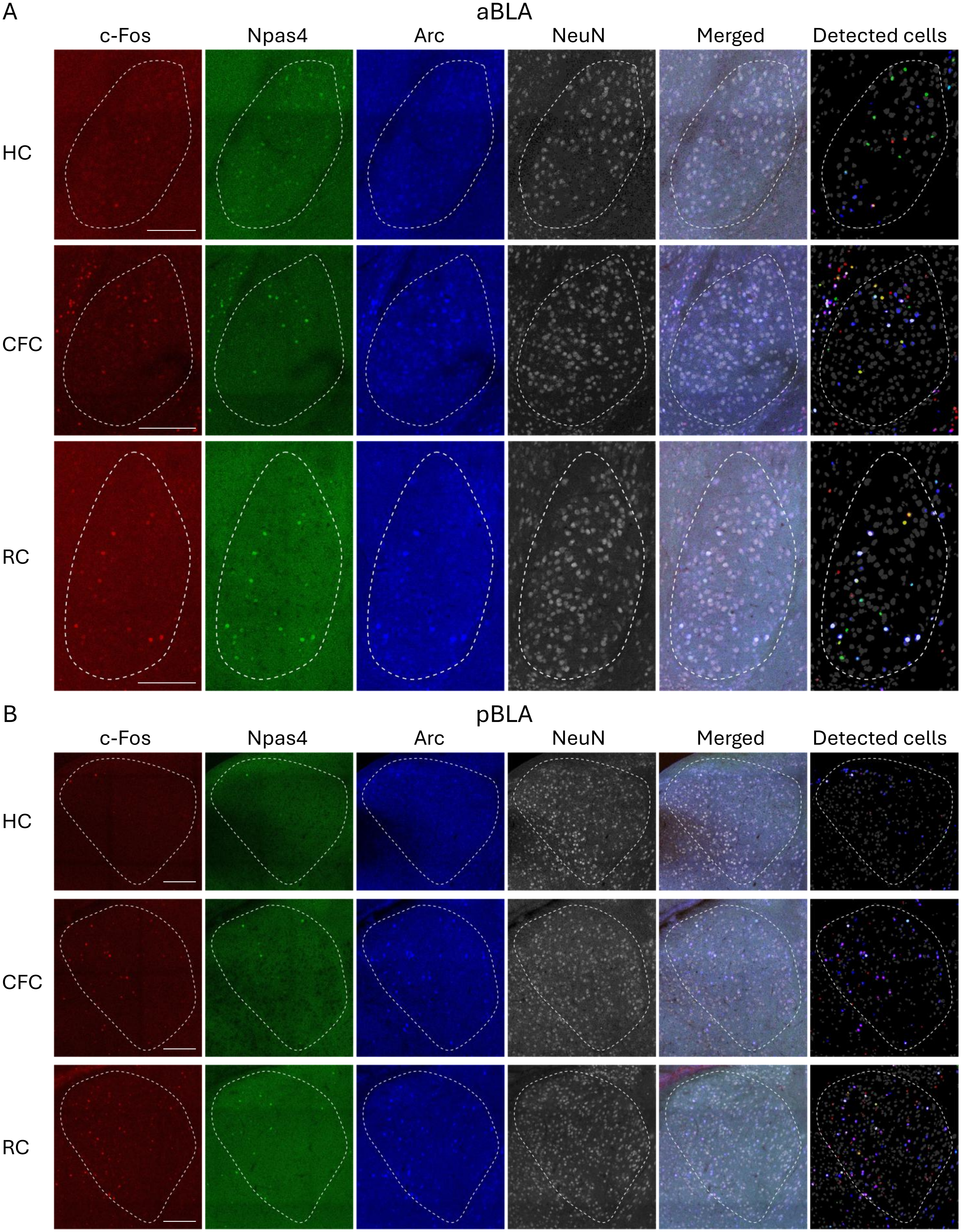

Supp. Figure S2: IEG expression in BLA

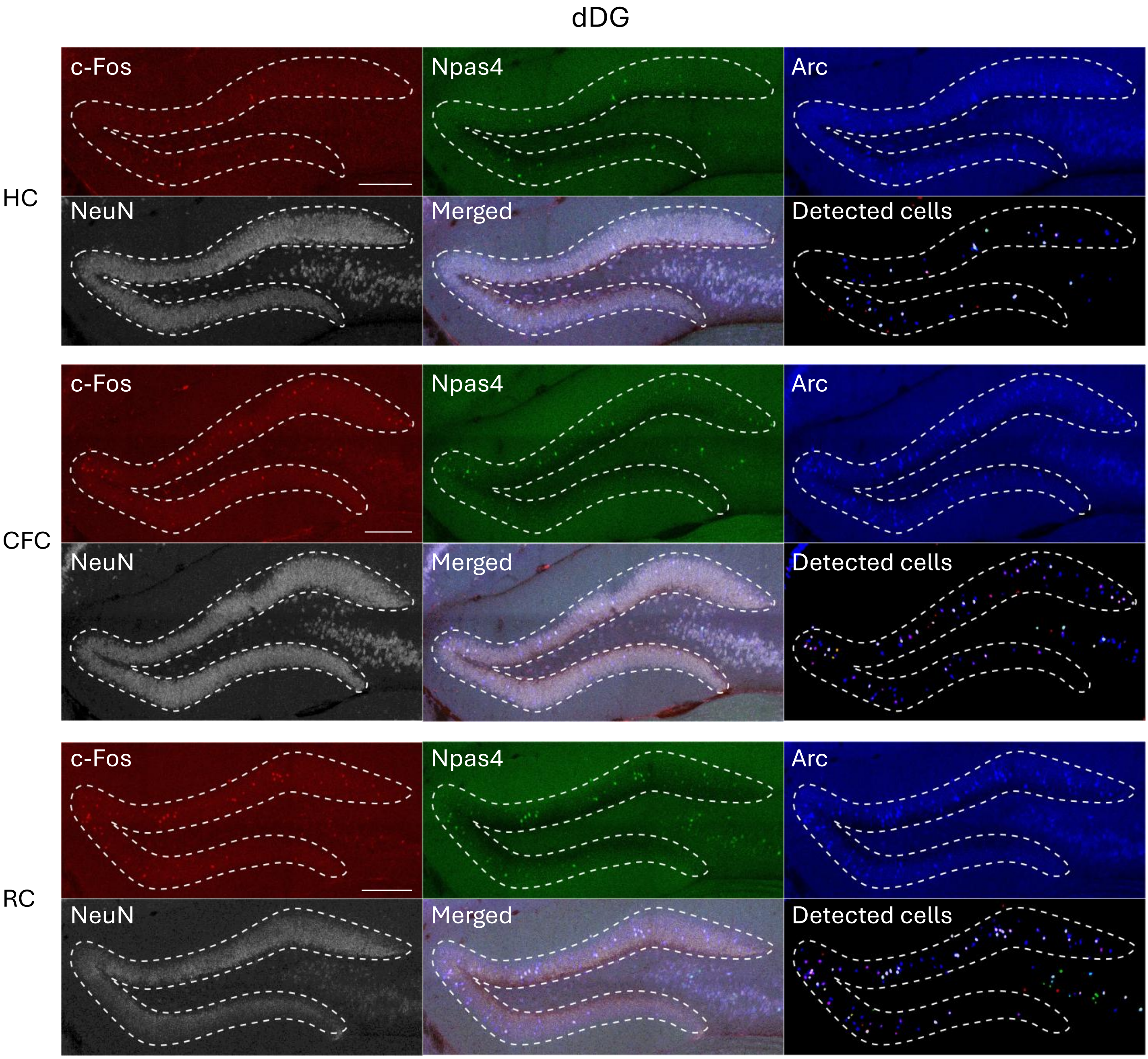

Supp. Figure S3: IEG expression in dDG

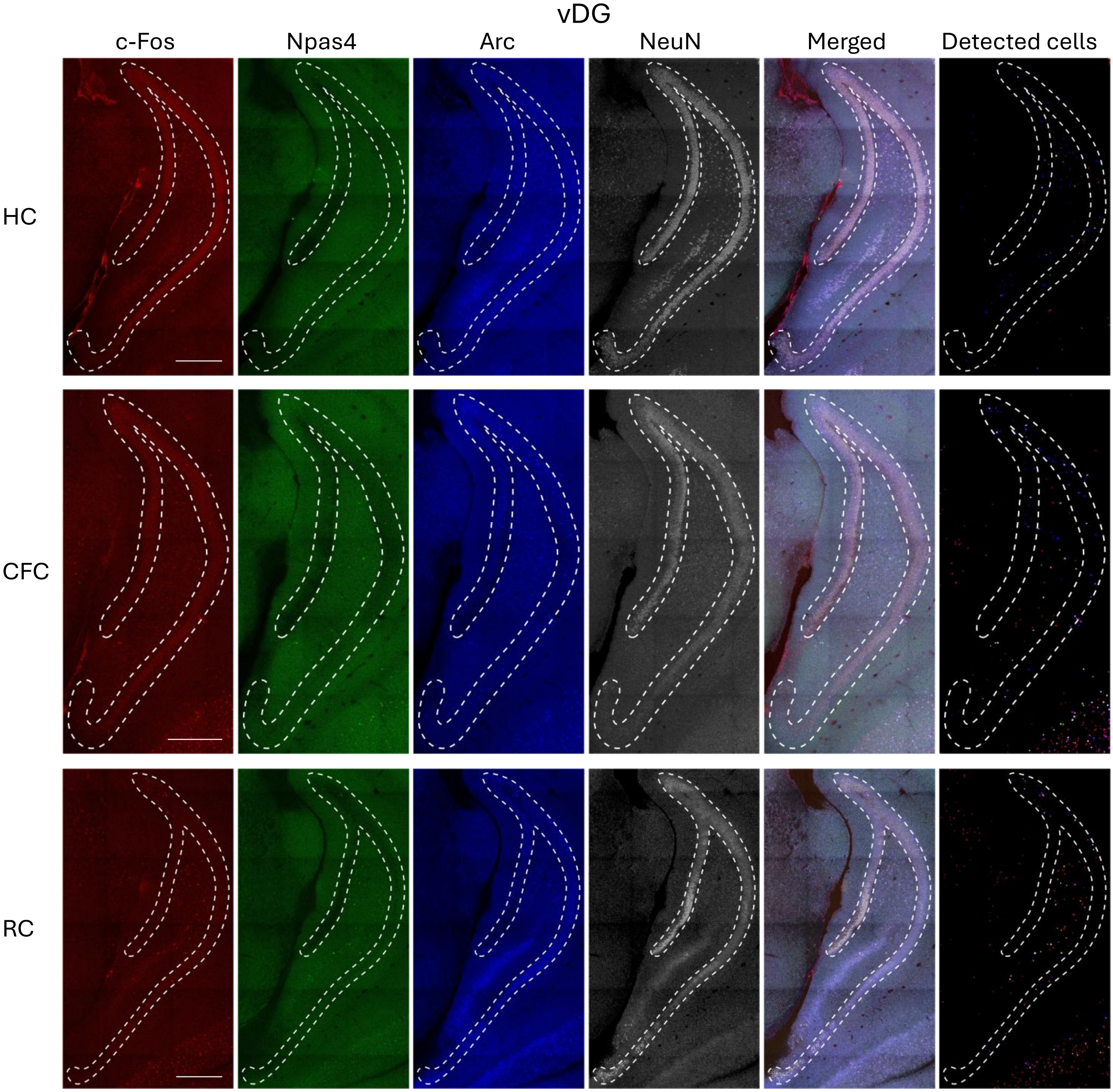

Supp. Figure S4: IEG expression in vDG

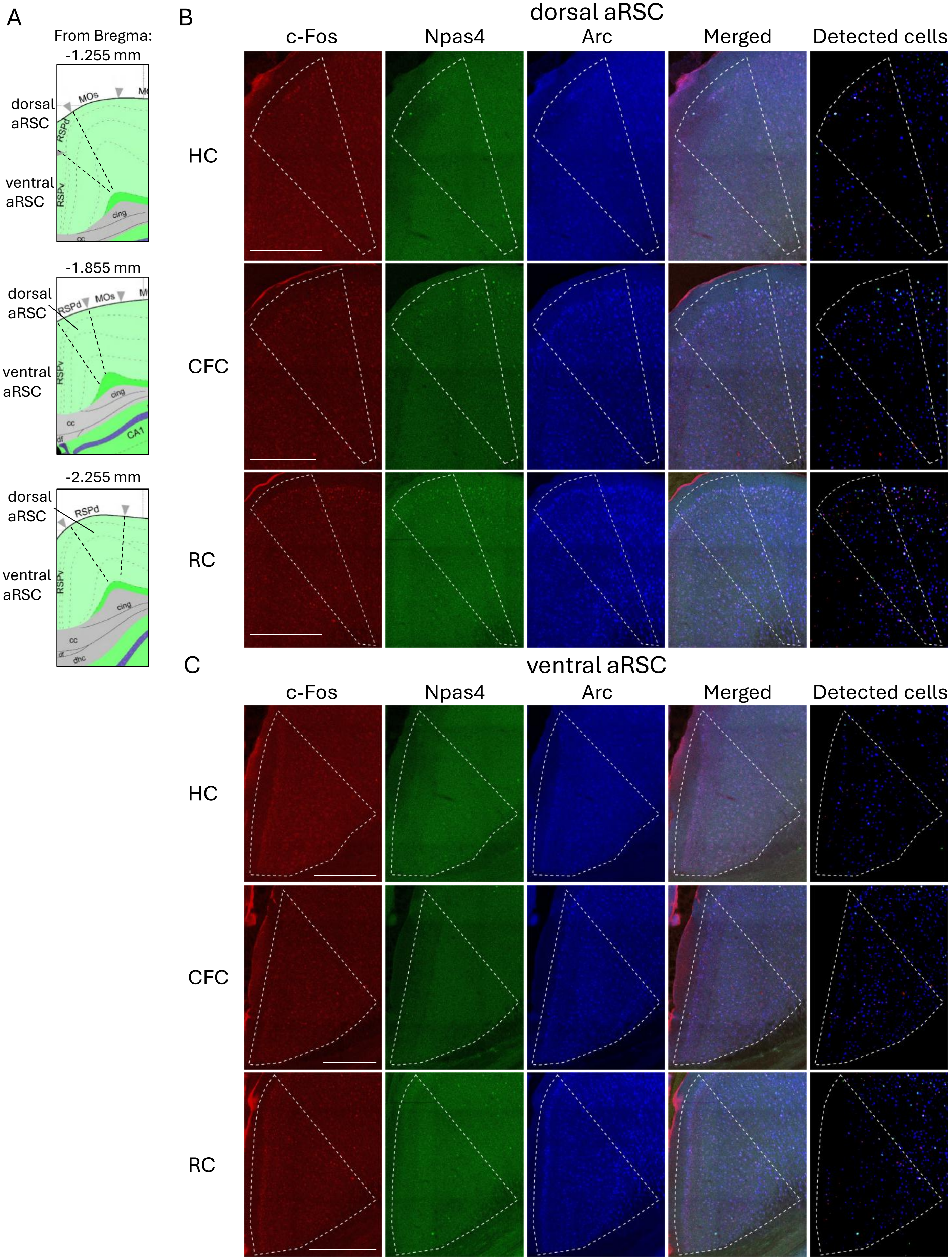

Supp. Figure S5: IEG expression in aRSC

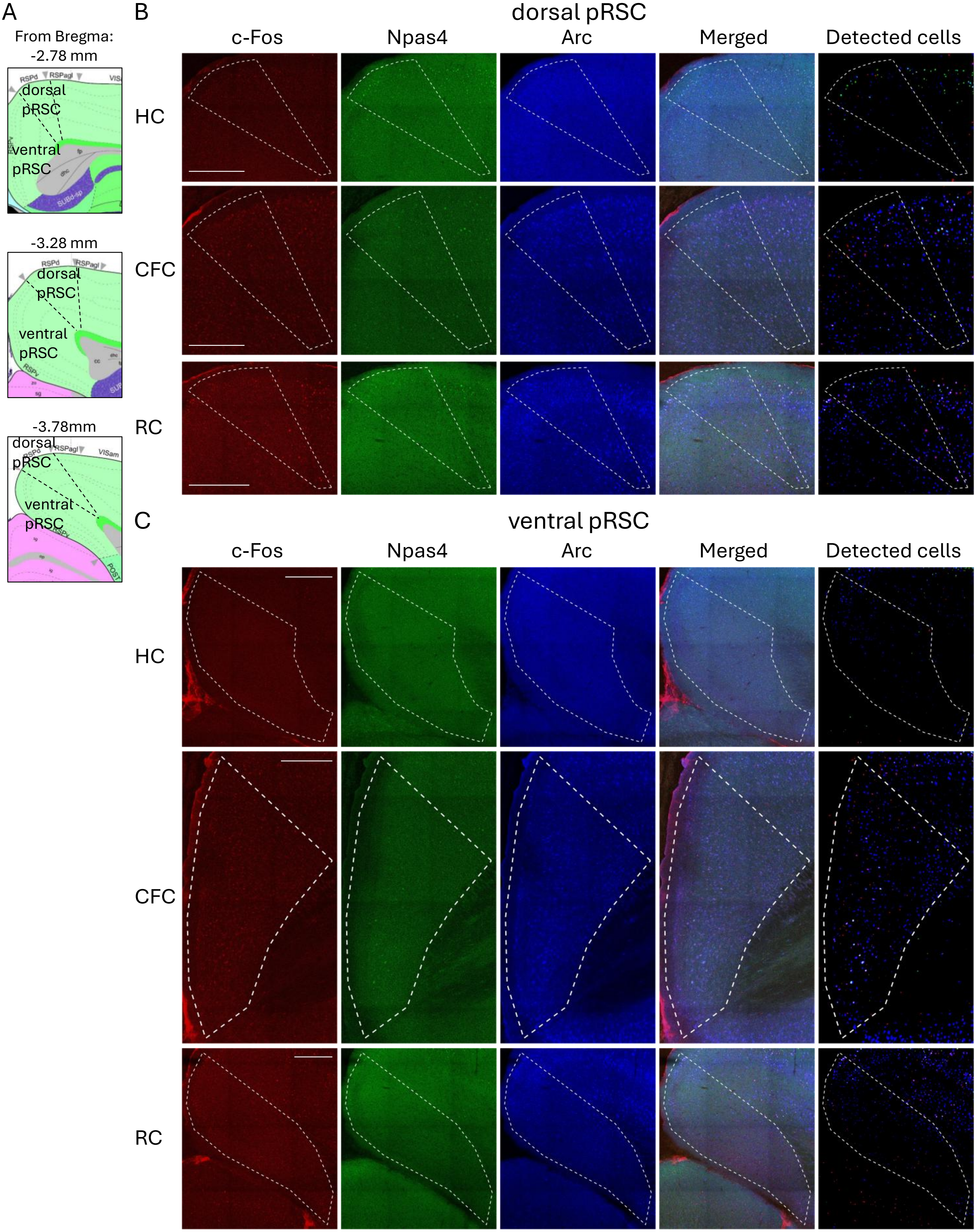

Supp. Figure S6: IEG expression in pRSC

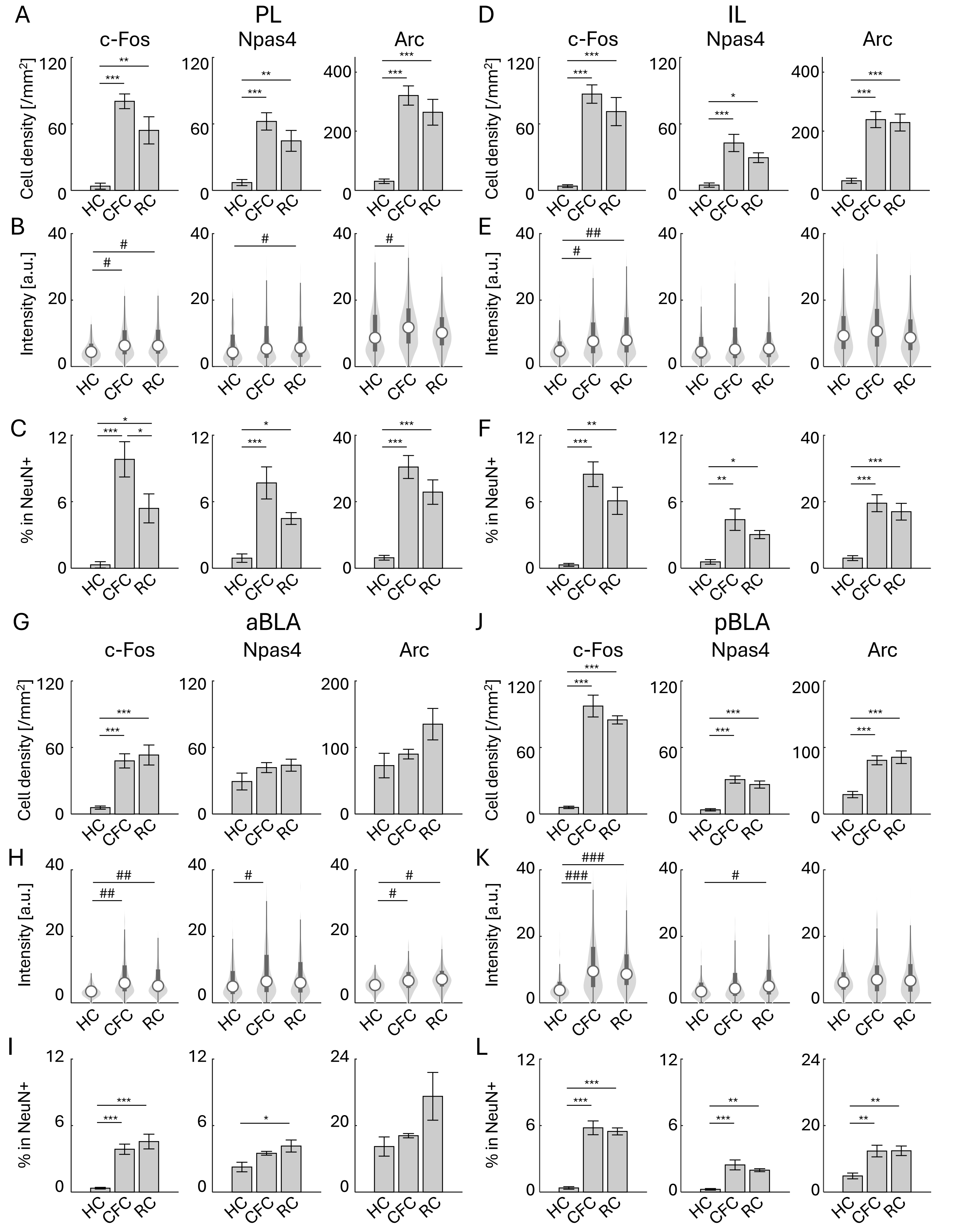

Supp. Figure S7: Cell density and expression level of IEG-positive cells in PFC and BLA

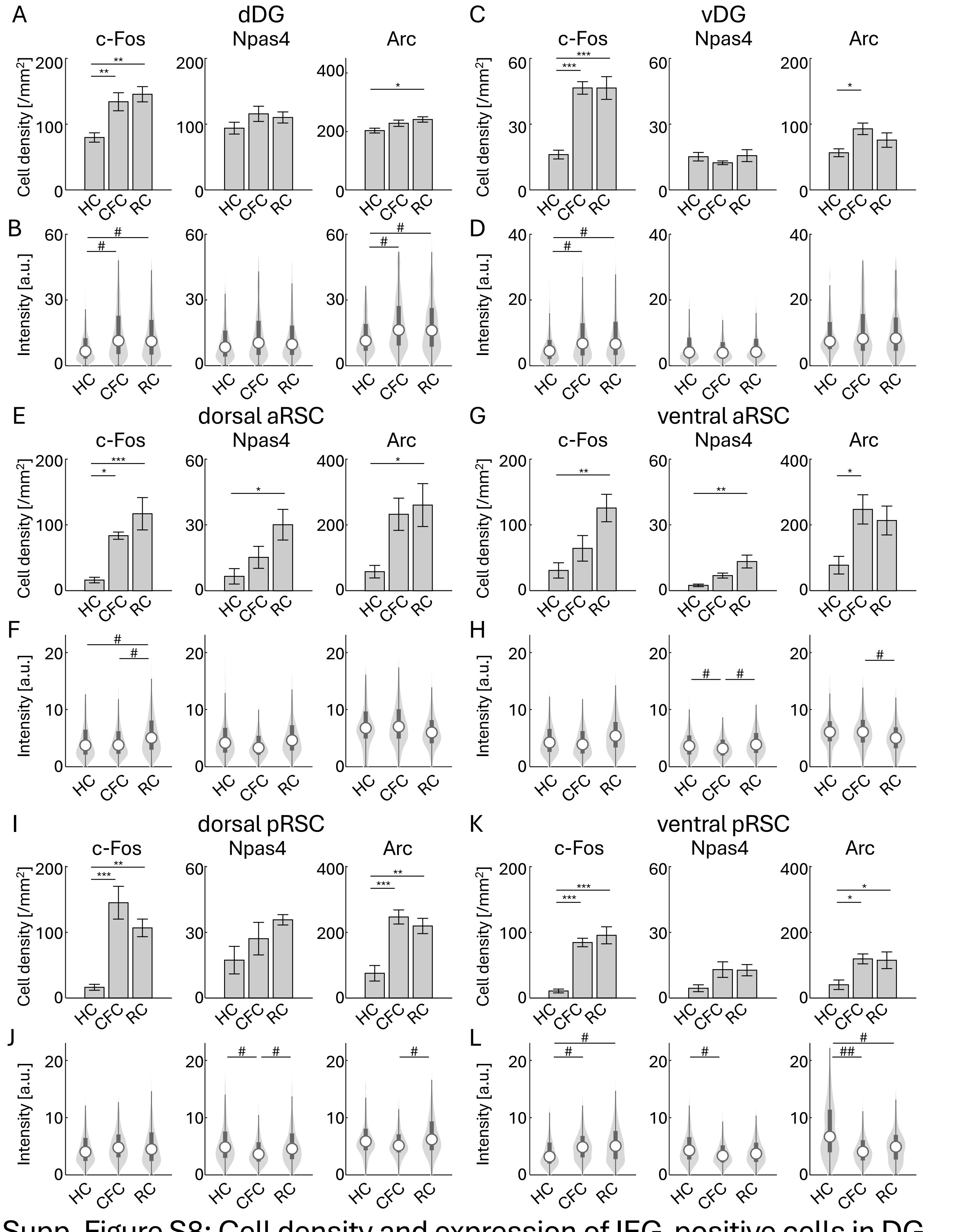

Supp. Figure S8: Cell density and expression of IEG-positive cells in DG and RSC

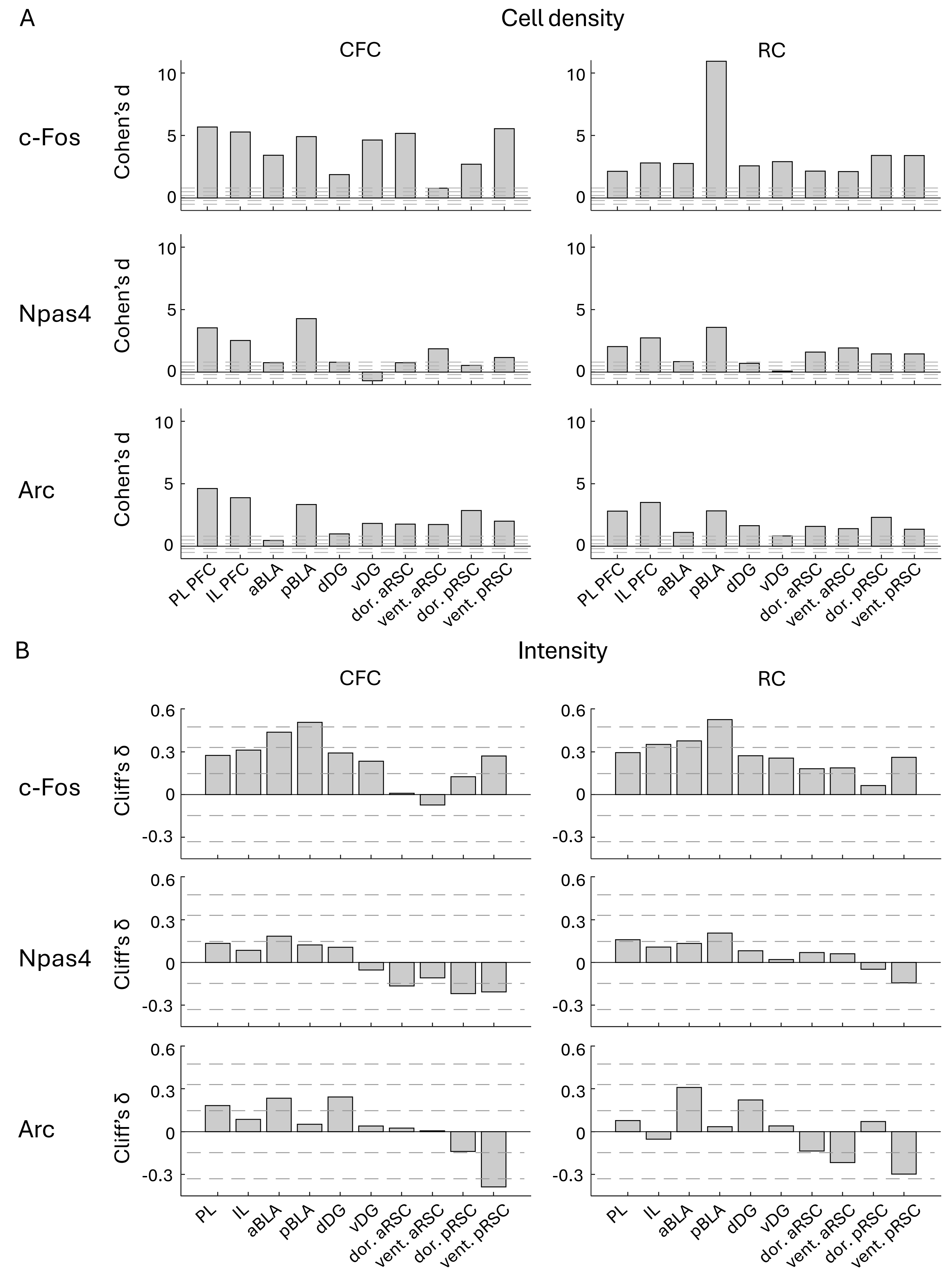

Supp. Figure S9: Effect size of cell density and intensity

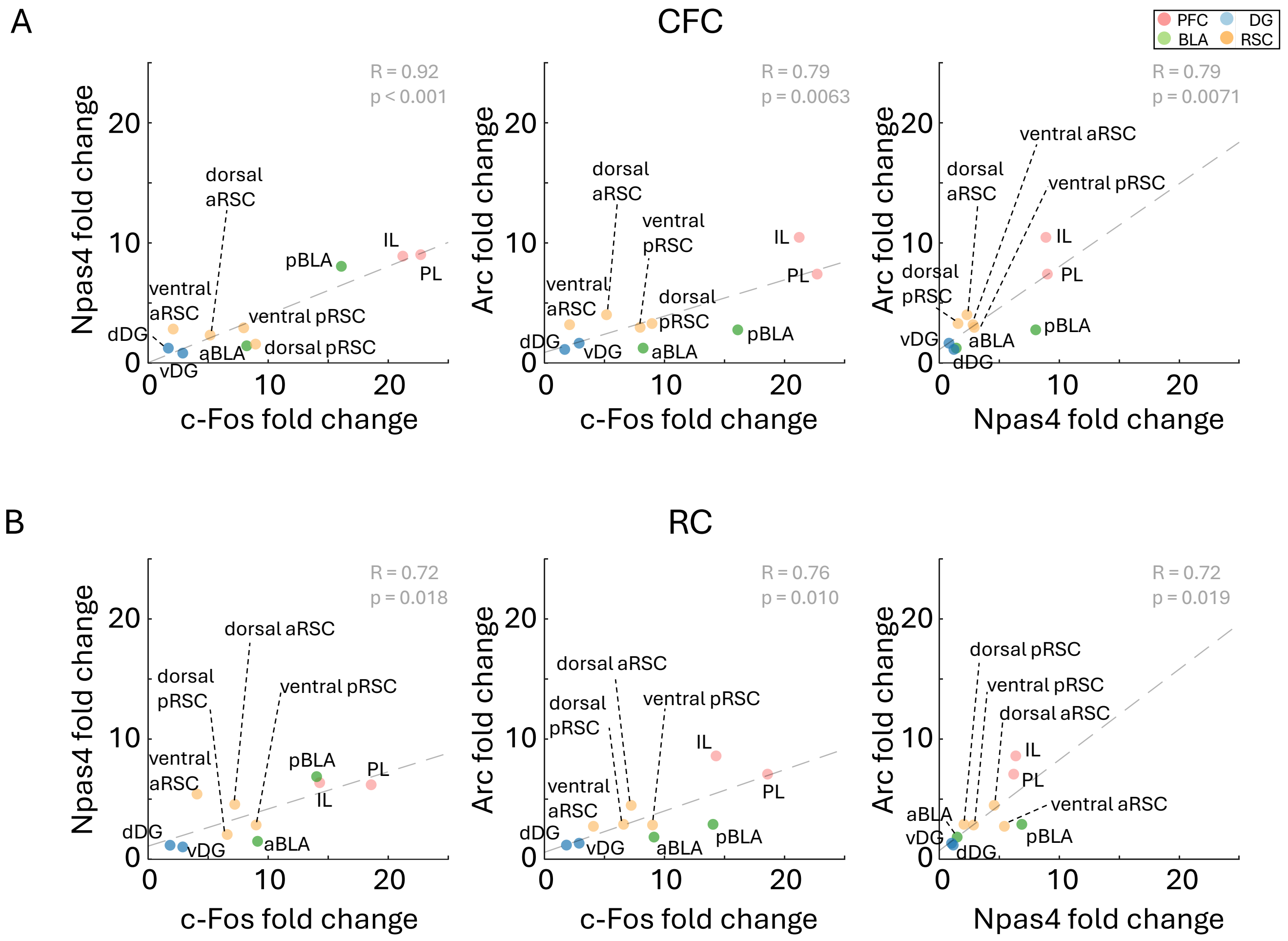

Supp. Figure S10: Cell density changes in each IEG in different brain regions

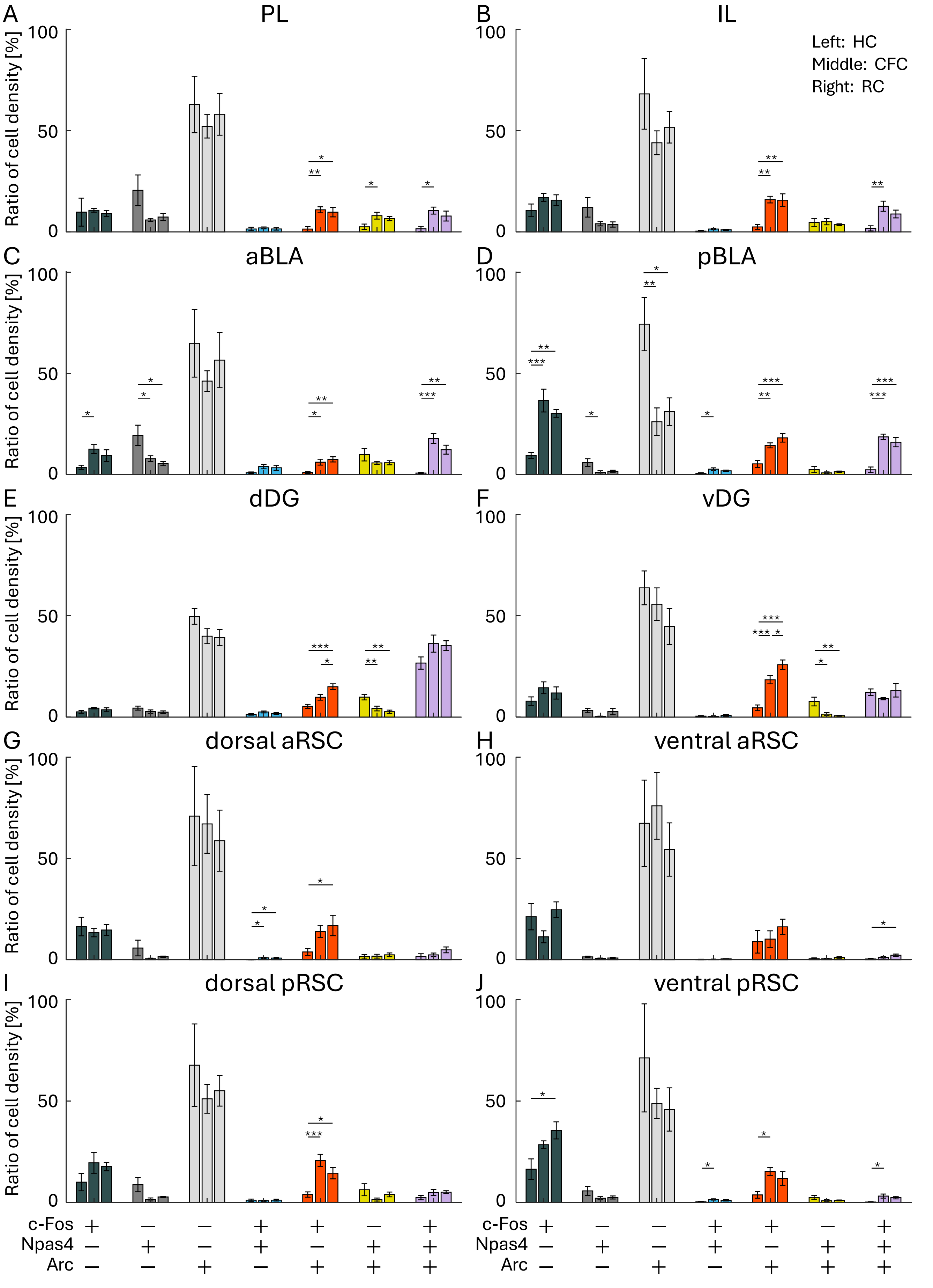

Supp. Fig. S11: Cell density ratio per all IEG-positive cells in each cell group

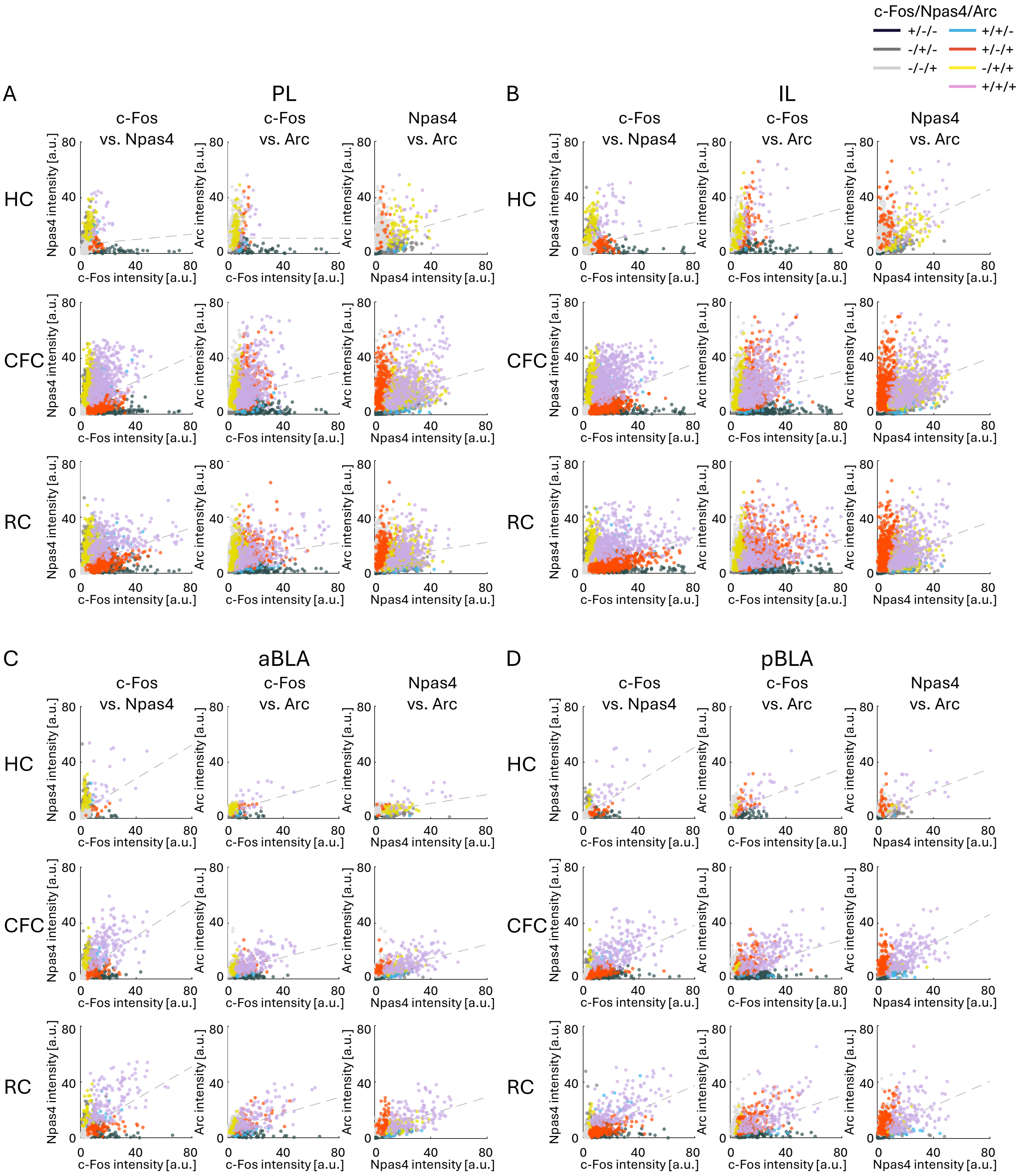

Supp. Figure S12: Intensities of IEGs in individual cells in PFC and BLA

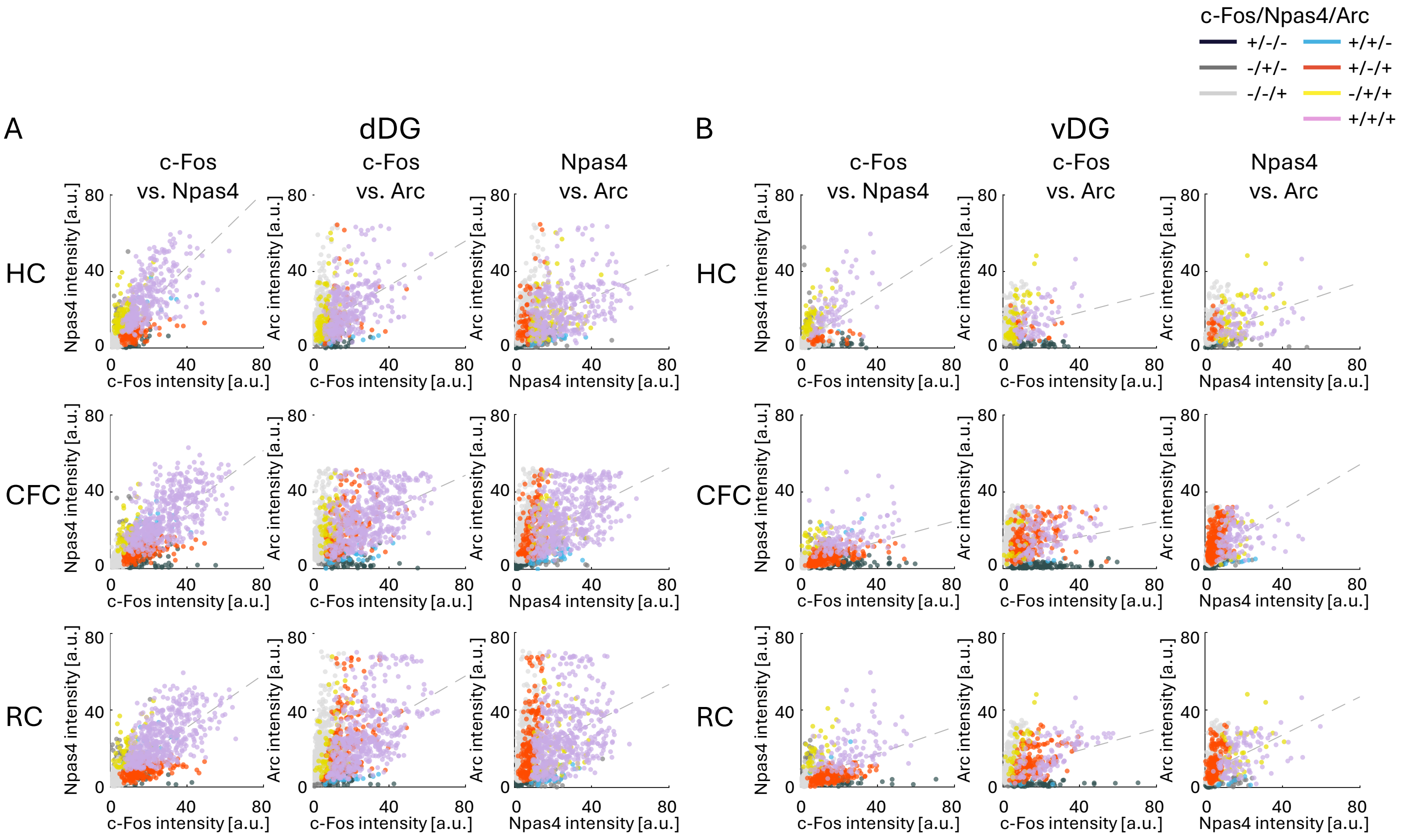

Supp. Figure S13: Intensities of IEGs in individual cells in DG

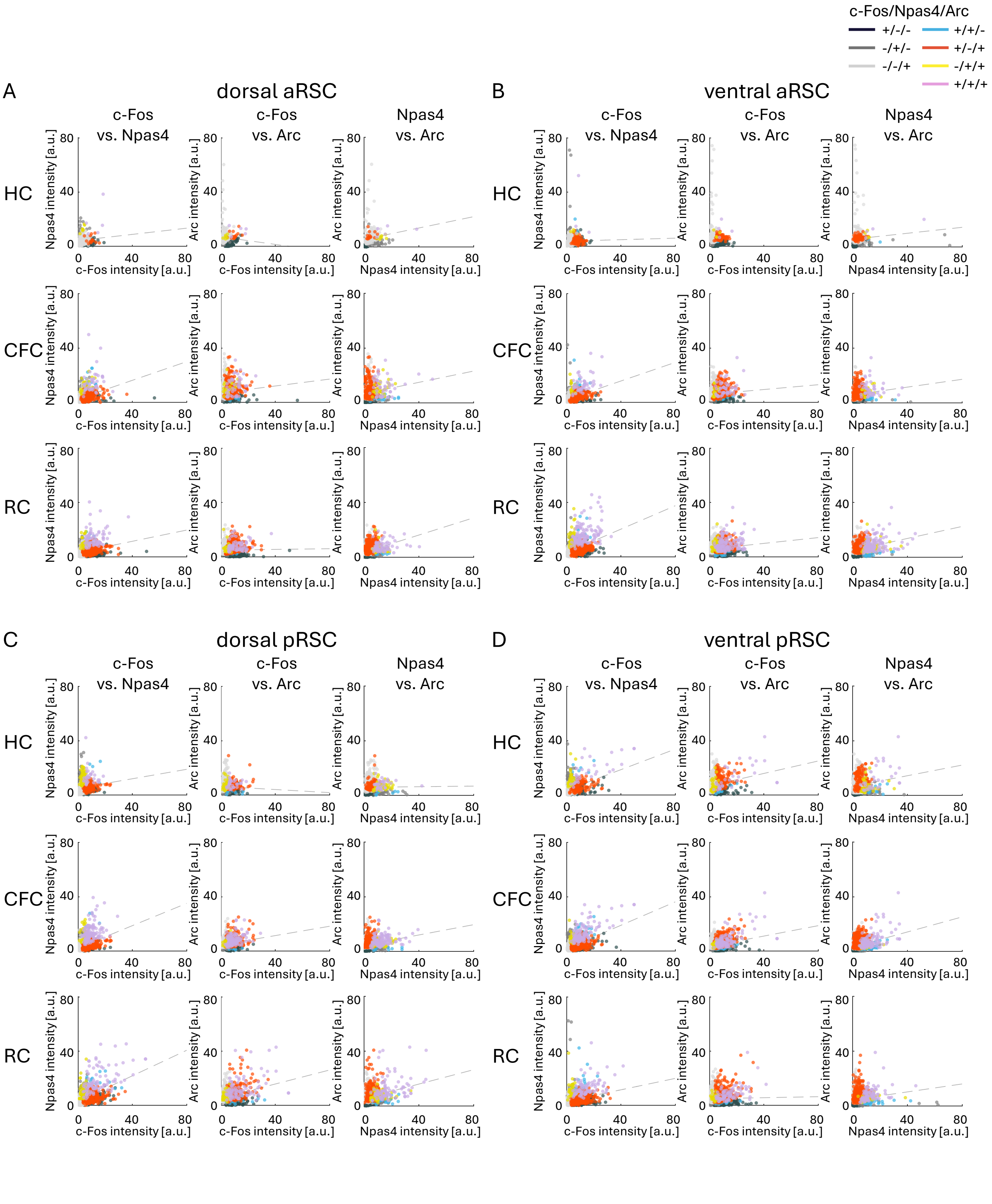

Supp. Figure S14: Intensities of IEGs in individual cells in RSC

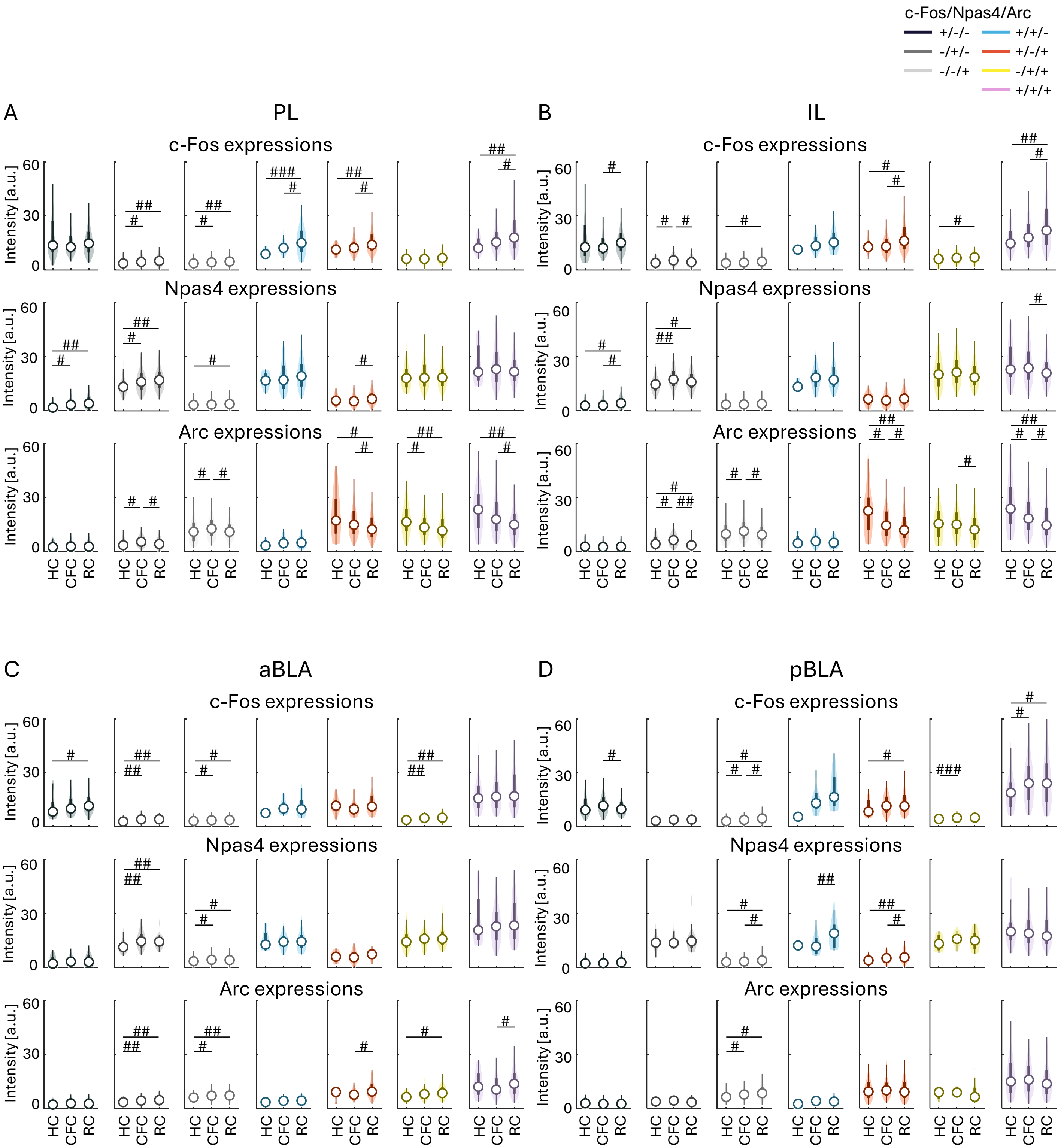

Supp. Figure S15: Intensities of IEGs in each cell group in PFC and BLA

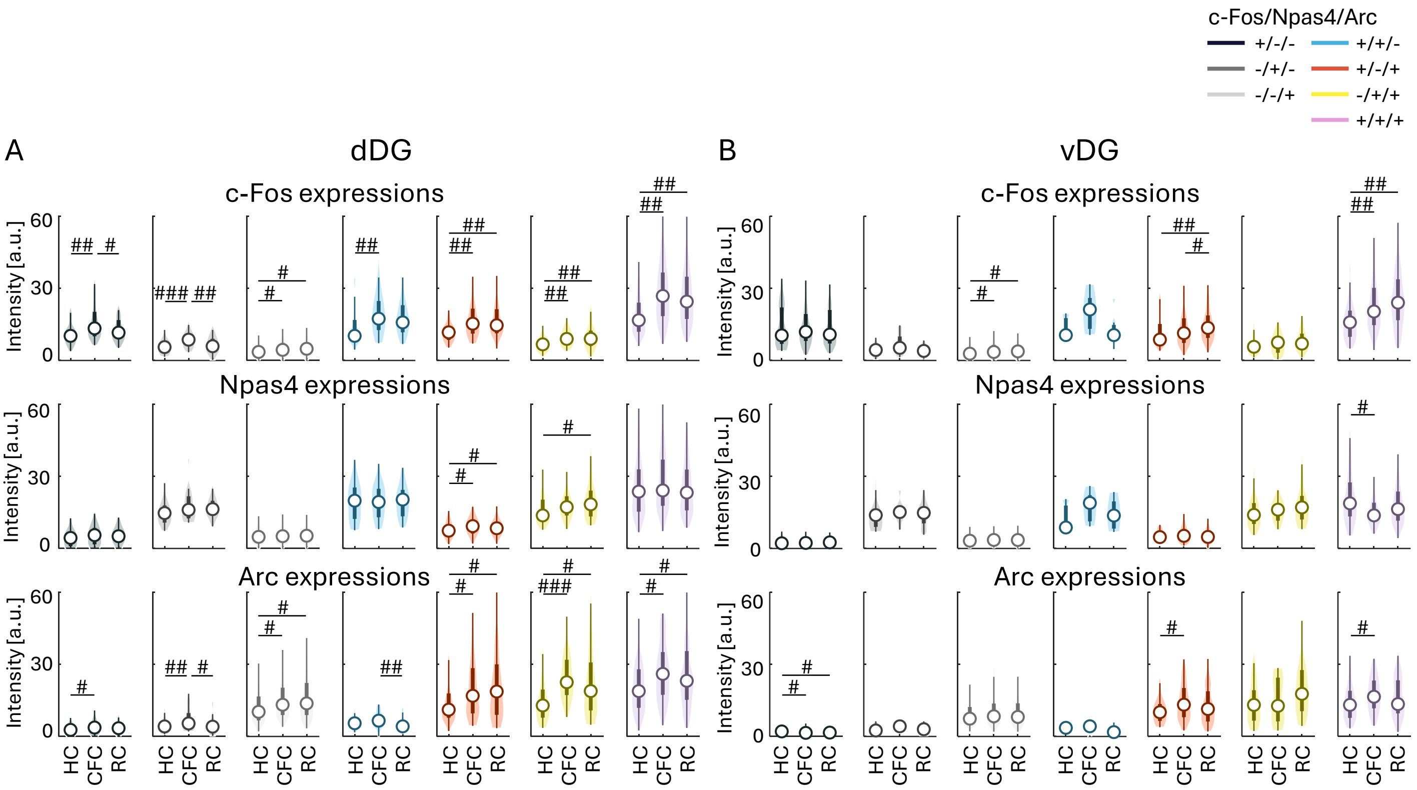

Supp. Figure S16: Intensities of IEGs in each cell group in DG

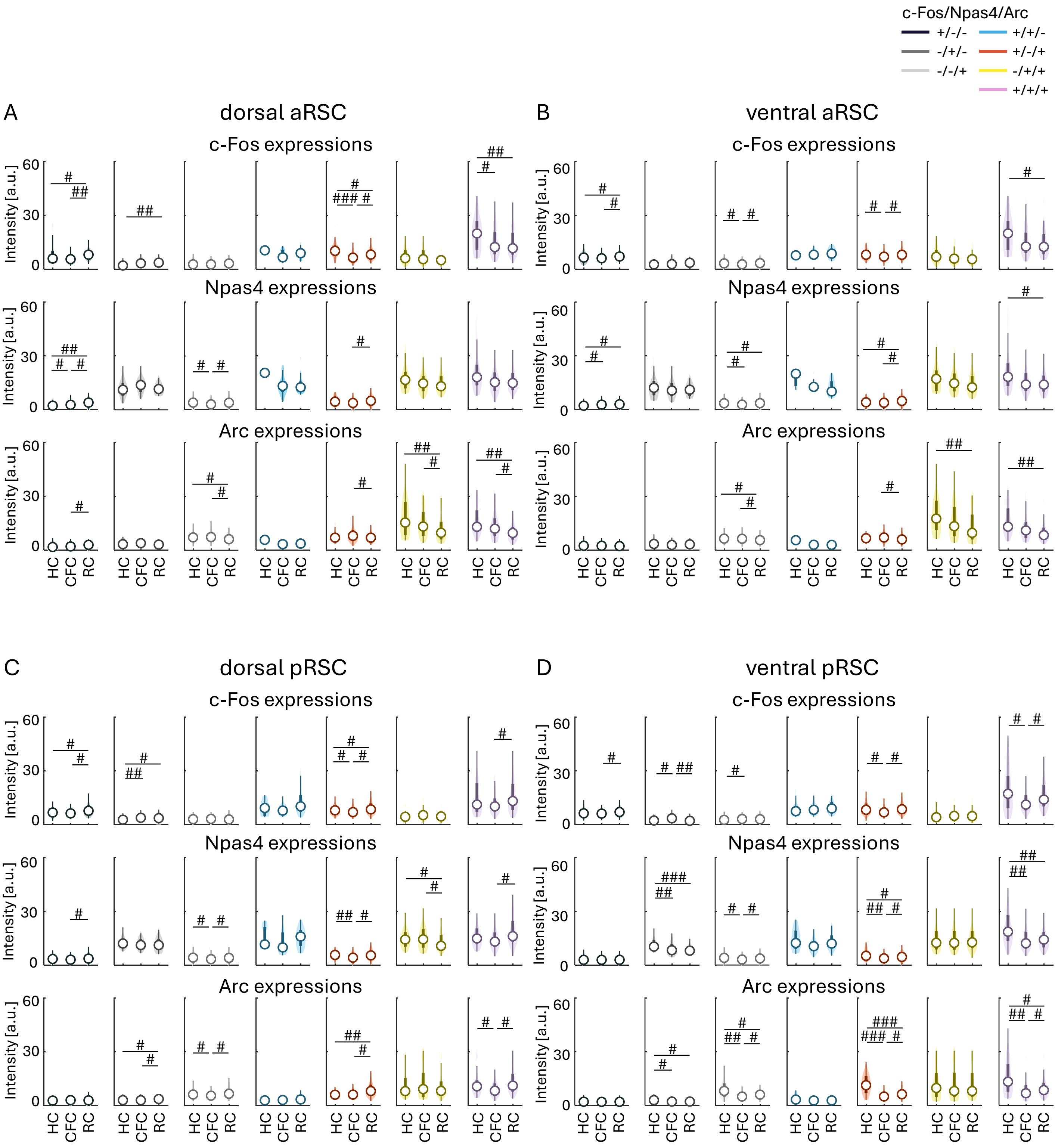

Supp. Figure S17: Intensities of IEGs in each cell group in RSC

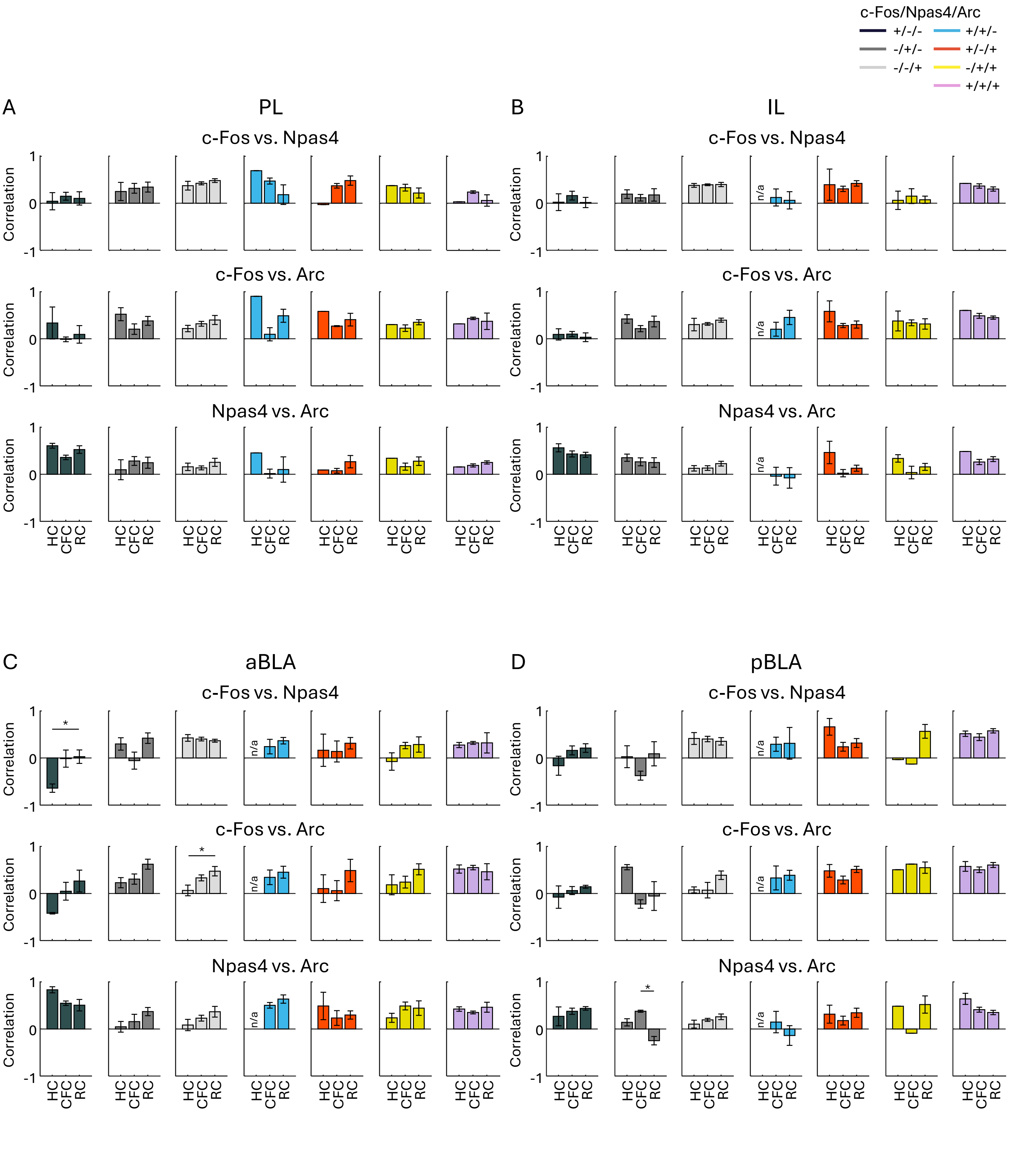

Supp. Figure S18: Intensities correlations in each cell group in PFC and BLA

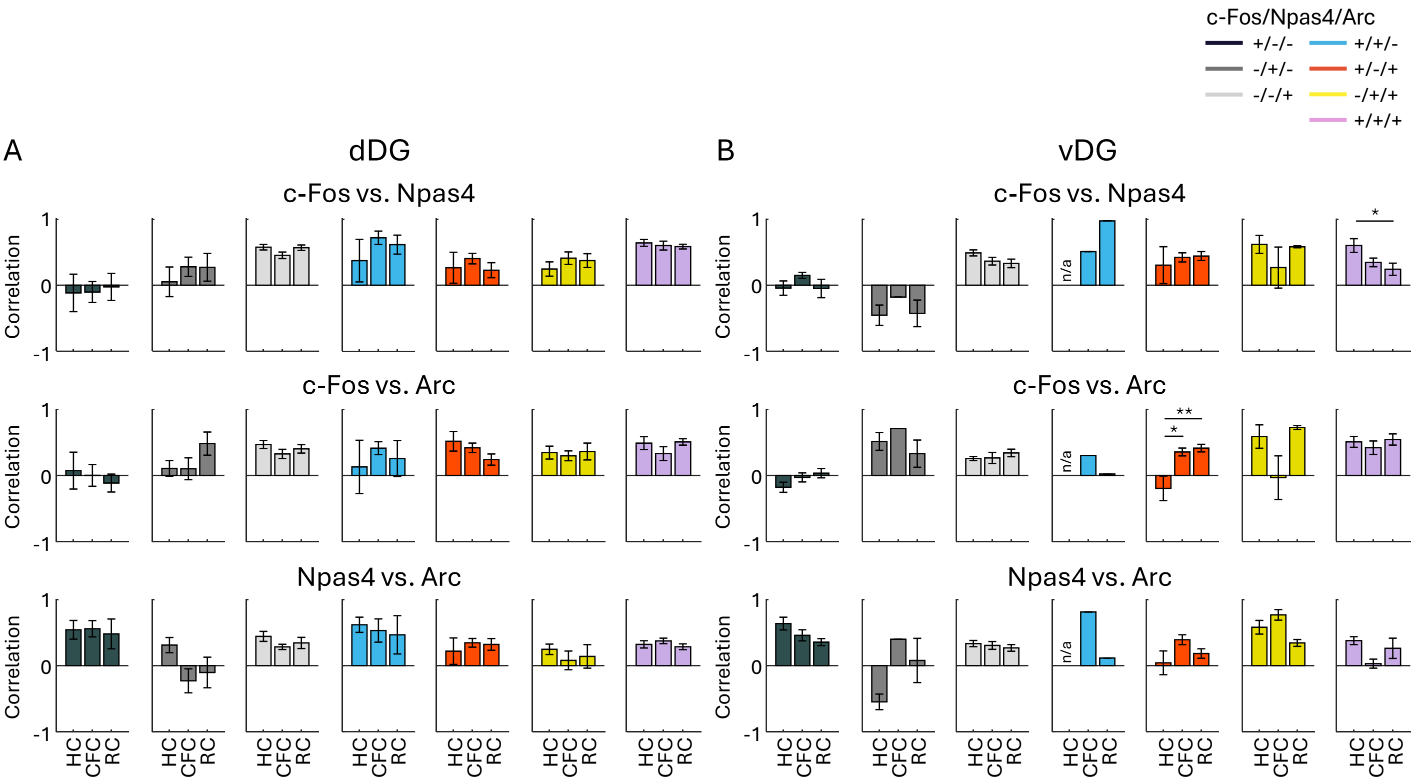

Supp. Figure S19: Intensities correlations in each cell group in DG

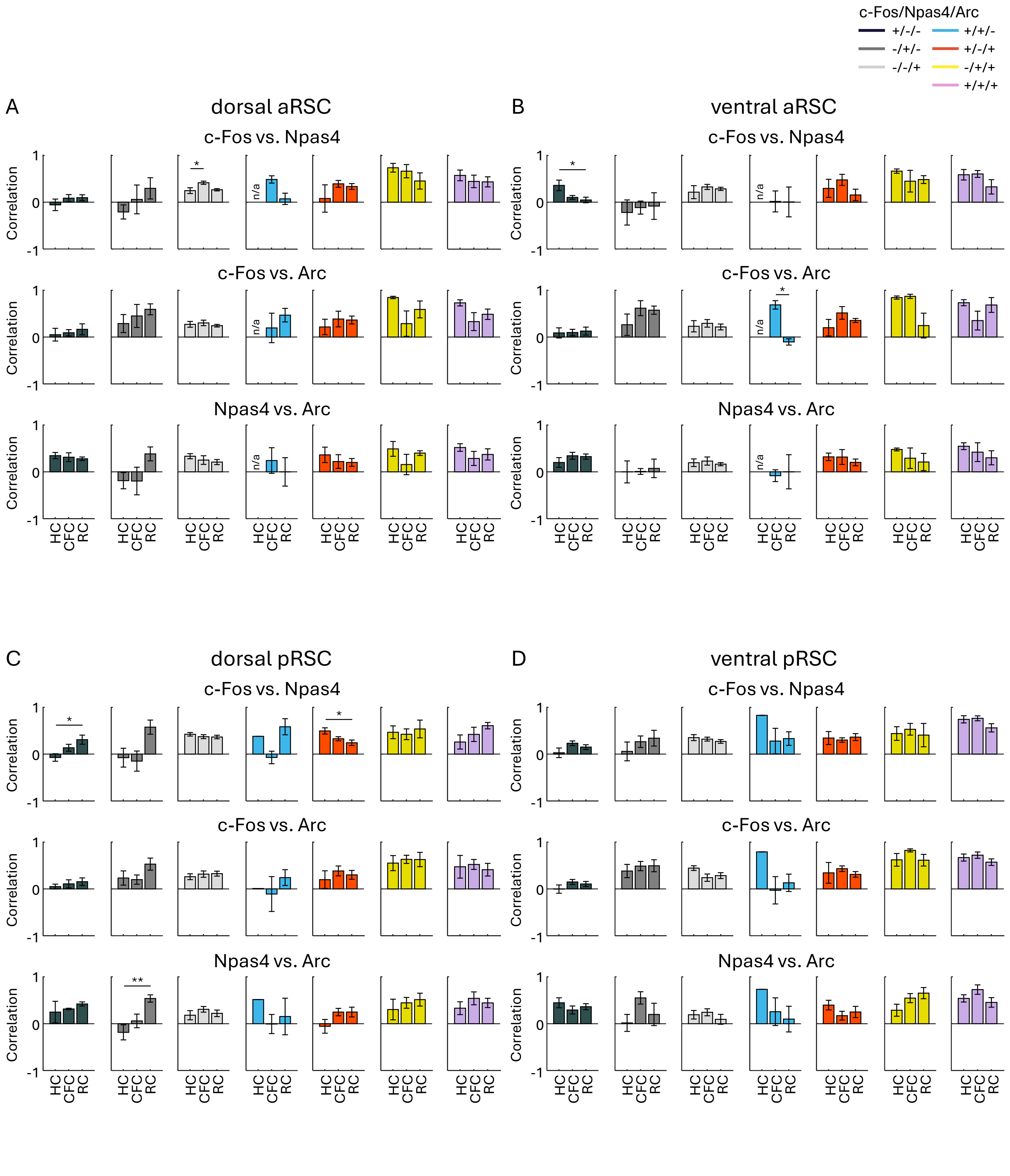

Supp. Figure S20: Intensities correlations in each cell group in RSC

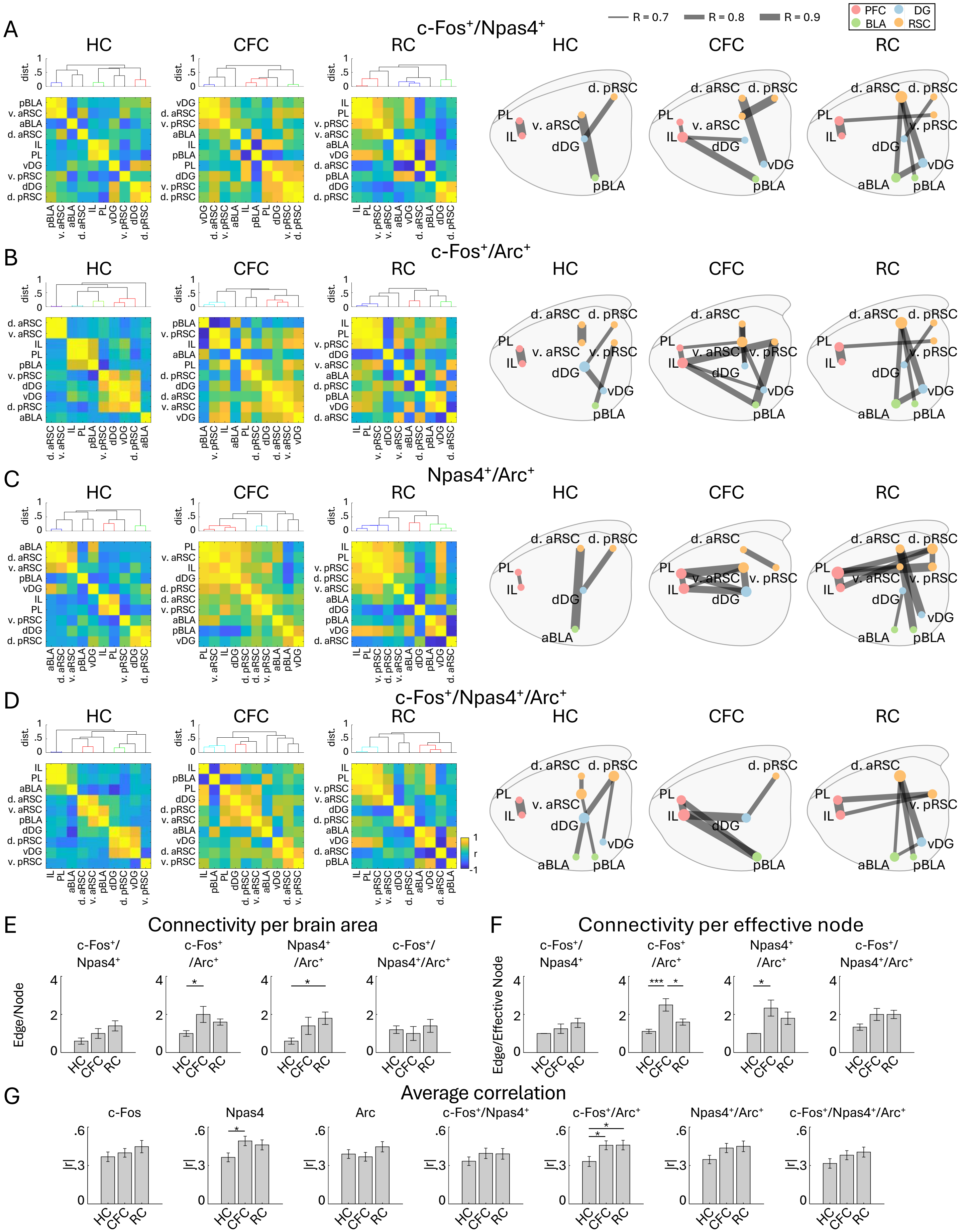

Supp. Figure S21: Functional connectivity network of IEG overlapping cells
